# Macroscopic rotation of photon polarization induced by a single spin

**DOI:** 10.1038/ncomms7236

**Published:** 2015-02-17

**Authors:** Christophe Arnold, Justin Demory, Vivien Loo, Aristide Lemaître, Isabelle Sagnes, Mikhaïl Glazov, Olivier Krebs, Paul Voisin, Pascale Senellart, Loïc Lanco

**Affiliations:** 1 Laboratoire de Photonique et de Nanostructures, CNRS UPR 20, Route de Nozay, 91460 Marcoussis, France; 2 Ioffe Physical-Technical Institute of the RAS, 194021 St-Petersburg, Russia; 3 Département de Physique, Ecole Polytechnique, F-91128 Palaiseau, France; 4 Université Paris Diderot—Paris 7, 75205 Paris CEDEX 13, France

## Abstract

Entangling a single spin to the polarization of a single incoming photon, generated by an external source, would open new paradigms in quantum optics such as delayed-photon entanglement, deterministic logic gates or fault-tolerant quantum computing. These perspectives rely on the possibility that a single spin induces a macroscopic rotation of a photon polarization. Such polarization rotations induced by single spins were recently observed, yet limited to a few 10^−3^ degrees due to poor spin–photon coupling. Here we report the enhancement by three orders of magnitude of the spin–photon interaction, using a cavity quantum electrodynamics device. A single hole spin in a semiconductor quantum dot is deterministically coupled to a micropillar cavity. The cavity-enhanced coupling between the incoming photons and the solid-state spin results in a polarization rotation by ±6° when the spin is optically initialized in the up or down state. These results open the way towards a spin-based quantum network.

## Abstract (web summary)

The recently observed rotation of a photon's polarization by interaction with a single solid state spin has potential implications in quantum computing. Here, Arnold *et al*. demonstrate enhanced spin–photon coupling and polarization rotation via a coupled quantum dot/micropillar cavity system.

## Graphical abstract

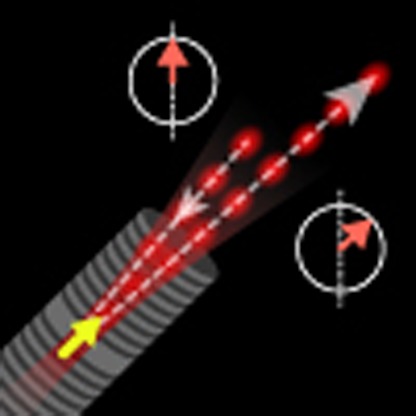

Solid-state spins hold many promises for quantum information processing^1^,^2^, and in the last decade a strong effort has been devoted to develop optical quantum operations with single spins in semiconductor quantum dots (QDs)^3^,^4^,^5^. Recently, spin–photon entanglement has also been demonstrated between a photon emitted by a quantum emitter and the spin degree of freedom of the same emitter^6^,^7^,^8^.

Another venue to spin–photon interfacing is to make use of the rotation of optical polarization (so-called Faraday or Kerr rotation) induced by a single spin placed at the centre of a cavity-quantum electrodynamics (QED) device. This approach allows interfacing a resident spin with a photon generated by an external source, opening new possibilities in quantum optics. A number of proposals have thus emerged^9^,^10^,^11^,^12^,^13^,^14^,^15^,^16^,^17^,^18^, which exploit large Faraday/Kerr rotations for various applications such as delayed photon–photon entanglement^9^, deterministic logic gates^10^ or fault-tolerant quantum computing^11^. However, although Faraday or Kerr polarization rotation in a magnetized medium is routinely used for magnetic material characterization^19^, observations of Kerr rotation induced by a single spin were reported only recently^20^,^21^,^22^, with rotation angles in the few 10^−3^ degree range.

In this work, we technologically implement a cavity-QED device coupled to a stationary spin qubit, allowing the enhancement by three orders of magnitude of the polarization rotation. A resident hole spin, in a semiconductor QD-pillar cavity device, is initialized^23^ and measured using resonant pump and probe beams: a Kerr rotation of several degrees is obtained. We finally show how quantum measurements and quantum entanglement can be implemented with realistic cavity-QED devices.

## Results

### Device characterization

**Figure 1:**
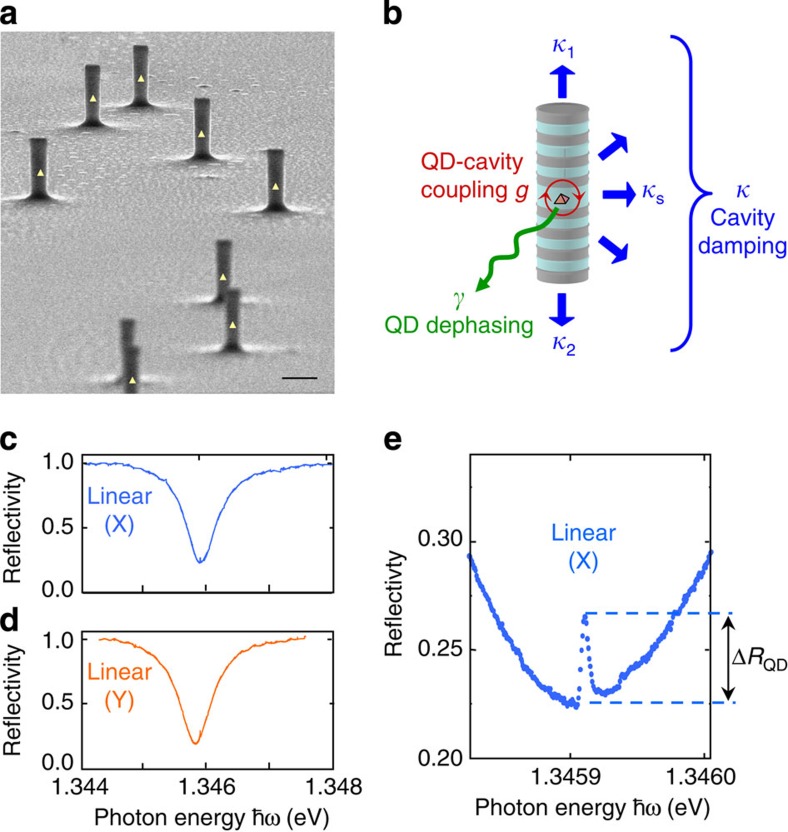
Quantum dot—pillar cavity device and its characterization by coherent reflection spectroscopy. (**a**) Scanning electron microscope view of various micropillar devices. Yellow triangles denote the positions of the deterministically coupled QDs. Scale bar, 5 μm. (**b**) Sketch of the QD micropillar device and corresponding interaction processes (red circle, internal QD-cavity coupling; blue arrows, cavity damping; green arrow, QD dephasing). (**c**,**d**) Device reflectivity as a function of the laser photon energy, for two orthogonal linear polarizations X and Y. The two modes are nearly polarization degenerate. (**e**) Zoom from **c**, focusing at the bottom of the reflectivity dip. The observed peak is a signature of a QD-induced transition resonant at 20 K with the X-polarized mode. The peak amplitude is referred to as the QD-induced reflectivity variation Δ*R*_QD_.

In the following, we study a single hole spin in a QD efficiently coupled to the mode of a micropillar cavity (Fig. 1a). Spectral and spatial matching between the QD transition and the pillar cavity mode are deterministically obtained using the *in-situ* lithography technique^24^ (see Methods). The fabricated device presents an optimal QD-cavity coupling strength, nearly polarization-degenerate optical modes and an efficient coupling with external beams^25^,^26^. The quantities describing this device (see Supplementary Note 1), sketched in Fig. 1b, are the QD-cavity coupling strength *g*, the QD field dephasing rate *γ* and the total cavity energy damping rate *κ=κ*_1_+*κ*_2_*+κ*_s_ (with *κ*_1,_ *κ*_2_ and *κ*_s_ the top mirror, bottom mirror and side-wall leakage rates, respectively). These parameters are combined into two figures of merit, the top mirror output coupling efficiency *κ*_1_/*κ* and the device cooperativity *C=g*^2^/*κγ*.

Preliminary device characterization is performed using coherent reflection spectroscopy^27^,^28^,^29^,^30^,^31^ (see Methods), using a continuous-wave (CW) laser with a finely tuneable angular frequency *ω* (1 MHz linewidth). The device reflectivity is displayed in Fig. 1c,d for two orthogonal polarizations (noted X and Y). For both polarizations, the reflectivity spectrum displays a Lorentzian cavity dip with *κ*=630 μeV full-width at half-maximum, corresponding to a quality factor *Q*=2,140. A small splitting between the two reflectivity dips is observed when comparing Fig. 1c,d. This is a signature of a residual pillar ellipticity, and the X and Y linear polarization directions correspond to the pillar minor and major axis. The mode splitting (90 μeV) is much smaller than *κ*; thus, the modes are close to polarization degeneracy. The top- and bottom-mirror output-coupling efficiencies are estimated to be *κ*_1_/*κ*=*κ*_2_/*κ*≈0.4 (see Supplementary Note 2) corresponding to a low side-wall leakage contribution *κ*_s_/*κ*≈0.2. This constitutes a substantial improvement compared with the device recently used to demonstrate a nonlinear optical response to few-photon pulses^25^, where (*κ*_1_+*κ*_2_)/*κ* was ~0.16. Finally, Fig. 1e displays a zoom centred on the X*-*polarized cavity dip, showing a narrow peak evidencing the efficient interaction with the QD transition: the resonantly excited dipole generates an optical field that interferes coherently with the exciting field. Furthermore, this transition corresponds to a QD which is charged with a resident hole (see Methods).

### Optical nonlinearity and hole spin initialization

**Figure 2:**
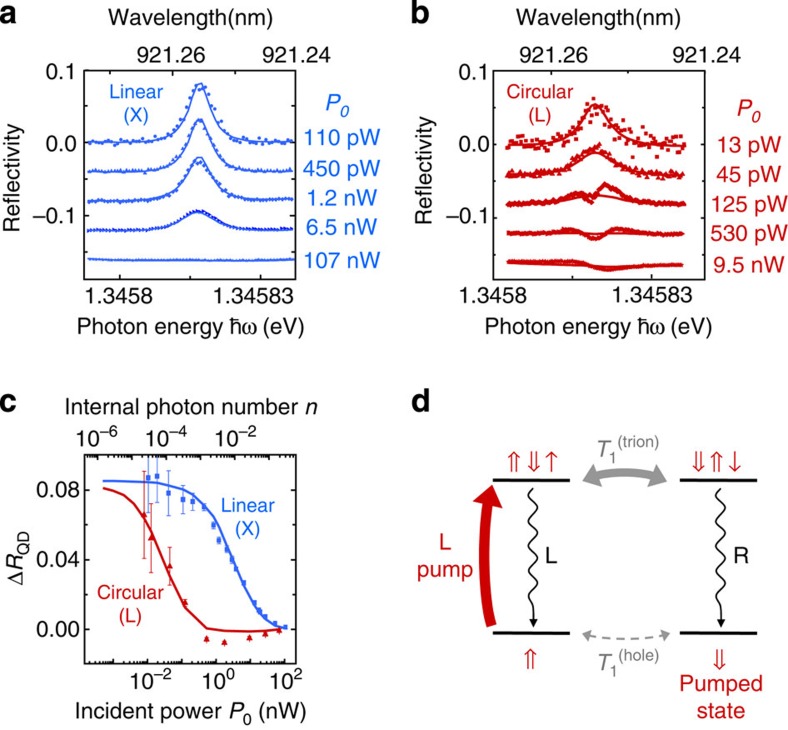
Optical pumping of a single spin. (In all panels, symbols are used for experimental measurements and solid lines for numerical simulations). (**a**,**b**) Reflectivity spectra centred on the QD-induced peak, for various values of the incident power *P*_0_, using linear (X) and circular (L) polarization, respectively. In both cases, a decrease of the peak amplitude is observed. The reflectivity spectra have been vertically shifted for clarity. (**c**) QD-induced reflectivity variations Δ*R*_QD_ measured for circularly and linearly polarized excitation, as a function of the incident power (bottom axis) and of the corresponding intracavity photon number *n* (top axis)^25^. (**d**) Four-level system and principle of hole spin pumping using a circularly polarized pump (ascending red arrow, resonant excitation with a left-handed (L) circularly polarized pump beam; black descending arrows, spontaneous emission of either L- or R-polarized photons; horizontal grey arrows, spin-flip processes).

Figure 2a presents several spectra, centred on the QD transition, measured with linearly (X) polarized excitation and for different incident powers *P*_0_. The QD resonance progressively disappears when the intracavity photon number increases in the cavity: this is the optical nonlinearity effect resulting from the saturation of the QD transition, as recently shown in ref. 25. Figure 2b displays similar spectra measured under left-handed (L) circularly polarized excitation, instead of a linearly (X) polarized excitation: a similar trend is observed, that is, a gradual disappearance of the QD peak with increasing power, yet for much lower powers. The QD-induced reflectivity variation, denoted Δ*R*_QD_, is defined as the absolute peak reflectivity subtracted by its value away from the QD resonance peak (see illustration in Fig. 1e): it is plotted in Fig. 2c as a function of the incident power *P*_0_. It shows that the threshold for the peak disappearance is two orders of magnitude lower for the circular polarization: as discussed below, this lower threshold is a signature that optical initialization of a resident-hole spin is occurring, as reported in ref. 23.

The hole ground state presents two spin configurations denoted |⇑› and **|**⇓›. The positive trion (two holes and an electron) formed after photon absorption is in either **|**⇑⇓⇑› or **|**⇓⇑⇓› state (**|**⇓› denotes the electron spin state). Whenever the hole is in state **|**⇑›, thanks to the polarization selection rules illustrated in Fig. 2d, optical pumping with L-polarized photons induces cycling transitions between the **|**⇑› and **|**⇑⇓⇑› states. Contact hyperfine interaction with nuclei may occasionally induce an electron spin flip leading to state **|**⇓⇑⇓›, and eventually to state **|**⇓› after spontaneous emission. As the hole spin-flip time *T*_1_^(hole)^ is much larger than the trion spin-flip time *T*_1_^(trion)^, this results in a stationary regime where the hole is initialized in state **|**⇓› (ref. 23). The QD peak then disappears from the reflectivity spectra, due to the **|**⇓›-**|**⇓⇑⇓› transition being transparent to incident L-polarized light. In summary, in circular polarization QD transparency is induced at low excitation power by the spin pumping effect, whereas in linear polarization it is induced by the saturation of both trion transitions, arising at higher excitation power.

As displayed in Fig. 2a–c, numerical simulations have been performed by solving the system master equation (see Supplementary Note 1). Our observations are well reproduced with a high coupling strength *g*=15 μeV and a low dephasing rate *γ*=2 μeV. This dephasing rate accounts for phonon and charge dephasing as pure-dephasing phenomena, and its value is consistent with the dephasing rate measured at 20 K in other works^30^,^32^. A ratio *T*_1_^(hole)^/*T*_1_^(trion)^=200 is obtained from the fit of the power difference between the disappearance thresholds under circular and linear polarization. These results show that despite a finite polarization splitting of the cavity mode, optical initialization in a given spin state is achieved. We note that some small deviation from theory is observed in Fig. 2b when the pump is set to circular polarization. This deviation might be a signature of a nuclear polarization induced by the circularly polarized pumping, an effect that is not taken into account in our model.

### Kerr rotation measurements

**Figure 3:**
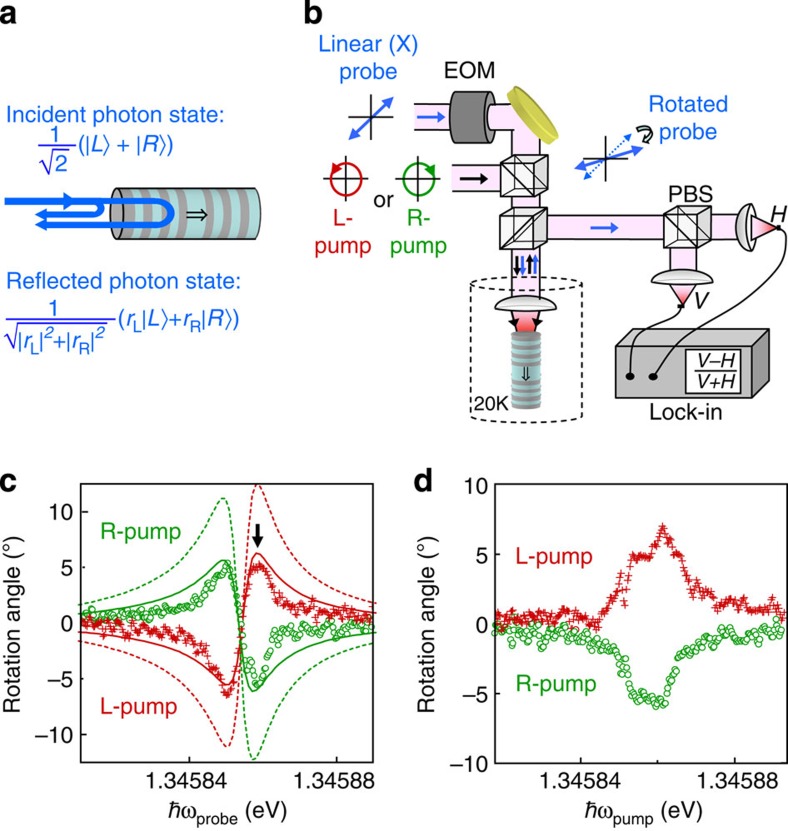
**Macroscopic Kerr rotation induced by a single spin in a cavity-QED device**. (**a**) Polarization states for the incident and reflected beam. The reflected beam results from the interference of two contributions: direct reflection and light injected into and re-extracted from the cavity. (**b**) Simplified scheme of the experimental setup used for spin optical pumping and Kerr rotation measurements on a QD-pillar cavity device. The polarization states are indicated: the probe beam (blue arrows) is linearly polarized, whereas the pump beam (black arrows) is either left-handed (L-pump) or right-handed (R-pump) circularly polarized. EOM, electro-optical modulator allowing the lock-in detection setup to filter out the contribution from the unmodulated pump beam. PBS, polarizing beam splitter, separating the horizontal (H) and vertical (V) components of the beam polarization, allowing the measurement of the Kerr rotation angle. (**c**) Kerr rotation angle as a function of *ω*_probe_, with *ω*_pump_ fixed at 1.345857, eV. Symbols: experimental data (L-pump in red, R-pump in green): macroscopic Kerr rotation angles up to +6° or −6° are obtained, depending on the pump polarization handedness. Solid line: theoretical fit with partial spin initialization. Dashed line: theoretical prediction with perfect spin initialization. (**d**) Kerr rotation angle as a function of *ω*_pump_, with *ω*_probe_ fixed at a maximum of Kerr rotation (thick vertical arrow in **c**).

In the following, we measure the Kerr rotation induced by the spin pumped either in the |⇑› or |⇓› state. To do so, a weak and coherent CW probe beam (*P*_probe_=110 nW, corresponding to *n*≈3 × 10^−4^ intracavity photons) is sent on the device. Its polarization is prepared in the linear state 
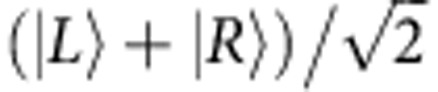
, where |*L*› and |*R*› are the photon states associated to the left-handed and right-handed circular polarizations, respectively. The reflected beam polarization is then in the normalized state 
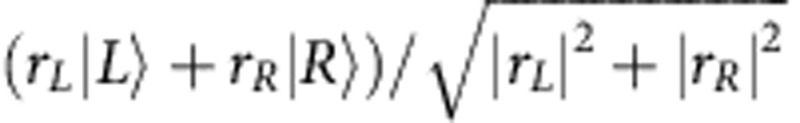
, with *r*_L_ and *r*_R_ being the complex reflection coefficients for L-polarized and R-polarized light (see Fig. 3a). When the hole is in state **|**⇓›, the left-handed component of the probe beam is insensitive to the QD transition, whereas the right-handed component experiences a reflectivity shift induced by the QD transition: thus, *r*_R_ depends on the detuning between the QD transition energy *ω*_QD_ and the probe photon energy *ω*_probe_, while *r*_L_ does not^10^,^12^. A symmetrical behaviour is expected when the hole is in state **|**⇑›. This results in a Kerr rotation of the probe beam polarization depending on the spin state. We note the corresponding output polarization states |Ψ_⇑_› and |Ψ_⇓_›, for the spin states **|**⇑› and **|**⇓› (see Supplementary Note 3).

The Kerr-rotation experimental setup is sketched in Fig. 3b. Two co-linear CW pump and probe beams, with photon angular frequencies *ω*_pump_ and *ω*_probe_, are focused on the upper surface of the micropillar. The pump beam polarization is chosen to be left-handed or right-handed, to initialize the spin in either the **|**⇑› or **|**⇓› state. The probe beam is linearly (X) polarized and modulated at 100 kHz. The reflected beams are sent onto a polarizing beam-splitter that separates the total reflected power into its horizontal and vertical components *P*_H_ and *P*_V_. These two components are measured with two avalanche photodiodes, and a lock-in amplifier filters the signal contribution arising from the probe. The Kerr rotation angle is then deduced from the measured photodiode contrast, *(P*_V_*−P*_H_*)/(P*_V_*+P*_H_). In the absence of polarization rotation, a zero contrast is observed. On the contrary, a positive (resp. negative) contrast will be observed in the case of a clockwise (resp. counter-clockwise) polarization rotation.

Figure 3c presents the Kerr rotation angle measured for both L-polarized and R-polarized pump beams, for a pump beam in resonance with the QD transition. A dispersive shape centred on *ω*_QD_ is observed for the Kerr rotation signal as a function of *ω*_probe_, as previously reported in ref. 20. However, here the Kerr rotation is macroscopic, with a maximum rotation angle of ±6°. This enhancement by three orders of magnitude is not simply explained by the multiple back-and-forth trips of the photons in the cavity, but results from an enhanced light–matter interaction in a cavity-QED regime. It requires a stringent control of the system: non-polarized cavity modes, precise positioning of the QD inside the cavity, low optical losses and low QD dephasing, together with a good optical coupling between the cavity mode and the incident laser beam. Quantitatively, two main features account for this huge enhancement. The first one is the increase of the spin–photon interaction induced by the optical confinement. This increase is governed by the device cooperativity *C=g*^2^/*κγ*≈0.2, which describes how efficiently a single two-level system modifies the optical properties of the confined mode. The second feature, specific to pillar-cavity devices, is the efficient interference between the directly reflected light and the light injected into and re-extracted from the cavity (see Fig. 3a). This interference is governed by the top-mirror output-coupling efficiency *κ*_1_/*κ*≈ 0.4.

The expected Kerr rotation angle is calculated using the QD and cavity parameters obtained from fitting the experimental data for spin pumping. The calculated rotation angle for perfect spin initialization fidelity is presented in dashed lines in Fig. 3c. The expected maximum rotation angle is 12°, twice larger than the experimental one, indicating an imperfect spin pumping in the pump–probe experiment. Indeed, to obtain a good signal-to-noise ratio, a probe power *P*_probe_=110 pW is used, representing 10% of the pump power *P*_pump_=1.3 nW. This leads to a non-negligible de-pumping of the spin state. A good agreement with the experimental data is obtained with a partial spin initialization, where the spin is in the desired state with a probability 0.75. As expected, the Kerr rotation is fully reversed when the pump polarization is set to right handed, corresponding to a spin pumping in the **|**⇑› state. Further evidence that this rotation arises from a single spin is obtained by measuring the Kerr rotation with varying *ω*_pump_, at a fixed *ω*_probe_. Figure 3d shows that the maximal Kerr rotation angle of ±6° is obtained for *ω*_pump_=*ω*_QD_, that is, when the optical spin pumping is the most efficient.

## Discussion

Our results show for the very first time that a macroscopic rotation induced by a single spin can experimentally be obtained: this is crucial to enable measuring a spin in a single-shot, non-destructive way, thanks to a very short measurement time. We now show that this enhancement can be pushed to the point where a single reflected photon, when detected by a photon counter, is sufficient to perform an ideal quantum non-demolition measurement.

**Figure 4:**
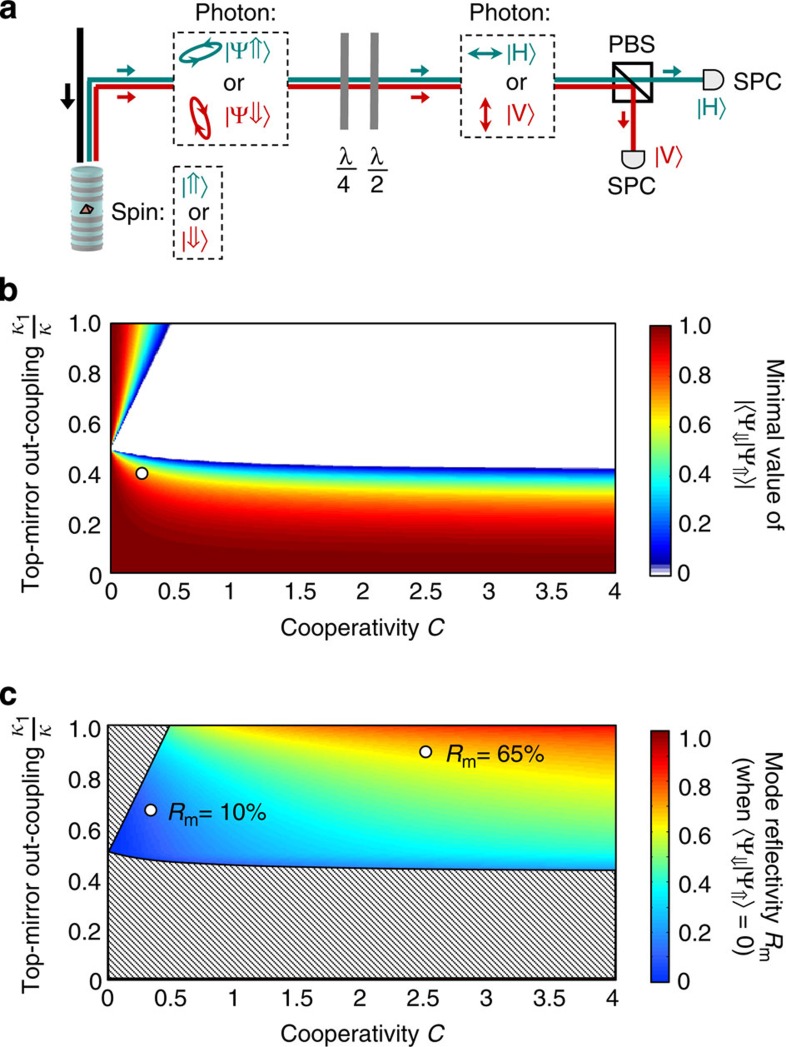
Towards spin-projective measurement with a single detected photon. (**a**) Principle of projective quantum-non-demolition measurement with a single detected photon, provided the two output polarization states are orthogonal (that is, ‹Ψ_⇓_|Ψ_⇑_›=0, with |Ψ_⇑_› and |Ψ_⇓_› the polarization states associated to the spin states **|**⇑› and **|**⇓**>**, respectively). In this experiment, a half-wave plate (*λ*/2) and a quarter wave-plate (*λ*/4) are used to perform unitary transformations mapping states |Ψ_⇑_› and |Ψ_⇓_> into states |H> and |V›. The latter are then distinguished using a polarizing beam-splitter (PBS) and single-photon counters (SPCs). (**b**) Minimal value of |‹Ψ_⇓_|Ψ_⇑_›| achievable for a given set of device parameters *C* and *κ*_1_ /*κ* (analytic calculations, see Supplementary Note 3): the ideal configuration ‹Ψ_⇓_|Ψ_⇑_›=0 can be obtained for a large range of parameters, represented by the white area. The parameters of the current device are represented by a white circle at *κ*_1_/*κ*=0.4 and *C*=0.2. (**c**) Maximal value of the mode reflectivity *R*_m_ achievable under the condition that ‹Ψ_⇓_|Ψ_⇑_›=0. The white circles indicate two sets of parameters (*κ*_1_/*κ*=0.66, *C*=0.3) and (*κ*_1_/*κ*=0.9, *C*=2.5). Increasing mode reflectivities are obtained when the device is further optimized. The hatched region corresponds to parameters for which the orthogonality condition can not be achieved.

An important figure of merit for quantum applications is the scalar product ‹Ψ_⇓_|Ψ_⇑_› between the two possible output polarization states, |Ψ_⇑_› and |Ψ_⇓_›, associated to the spin states **|**⇑› and **|**⇓›. This scalar product governs the level of measurement quantum back-action induced by the detection of a single-photon on the single spin state, that is, how strongly a single-photon detection event can project the spin in either the **|**⇑› or **|**⇓› state. The ideal case ‹Ψ_⇓_|Ψ_⇑_›=0 allows a maximal quantum back-action to be obtained, with a single-photon detection event leading to complete spin projection. Indeed, as sketched in Fig. 4a, if |Ψ_⇑_› and |Ψ_⇓_› are orthogonal they can be mapped into horizontal (|H›) and vertical (|V›) polarization states, which can then be unambiguously distinguished using single-photon detectors. In such an experiment, a single click on one single-photon counter will project the spin in state **|**⇑›, while a single click on the other one will project the spin in state **|**⇓›; this would constitute a projective quantum measurement performed with a single detected photon.

We also point out that the spin state can be initialized in a well-defined coherent superposition of **|**⇑› and **|⇓›**, using microwave pulses^33^ or optical pulses^3^ with a temporal profile adapted to the cavity lifetime^25^. A maximally entangled state 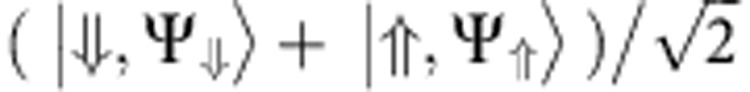 could then be produced between the spin and the reflected photon. As with the recent demonstrations of spin–photon entanglement^6^,^7^,^8^, these quantum operations would be conditioned on the actual detection of the entangled photon. Here the success probability of the entangling gate would be limited by the probability for the incident photon to be reflected, in other words, by the mode reflectivity *R*_m_. As discussed below, state orthogonality ‹Ψ_⇓_|Ψ_⇑_›=0 can be achieved with realistic devices together with large values of the success probability.

For each device, there is a minimal value of |‹Ψ_⇓_|Ψ_⇑_›|, which can be achieved through a proper choice of the QD-cavity detuning and probe photon energy. Figure 4b displays the calculated minimal value of |‹Ψ_⇓_|Ψ_⇑_›| achievable as a function of the cooperativity *C* and top-mirror output-coupling *κ*_1_/*κ*.; the current device with *κ*_1_/*κ*≈0.4 and *C*=0.2 is indicated by the white circle. What Fig. 4b shows is that the ideal situation ‹Ψ_⇓_|Ψ_⇑_›=0 can be reached for a large range of realistic values of *C* and *κ*_1_/*κ*. A single-sided cavity with *κ*_2_=0 (highly reflective bottom mirror), but with the present device values for *κ*_1_, *κ*_s_, *g* and *γ*, would correspond to *κ*_1_/*κ*≈0.66 and C≈0.3: this would be enough for achieving ‹Ψ_⇓_|Ψ_⇑_›=0, and thus maximal-fidelity quantum measurement and quantum entanglement operations using the reflected photons.

Finally, as shown in Fig. 4c, the ideal situation ‹Ψ_⇓_|Ψ_⇑_›=0 can be achieved together with a significantly high value of the mode reflectivity *R*_m_. Figure 4c displays the maximal mode reflectivity *R*_m_, which can be obtained as a function of *C* and *κ*_1_/*κ*, under the condition that ‹Ψ_⇓_|Ψ_⇑_›=0. It shows that the further a device is from the hatched region, the higher the achievable reflectivity. With a device for which *κ*_1_/*κ*≈0.66 and C≈0.3, this state orthogonality would be achieved with a mode reflectivity *R*_m_=10%. Increasing the cooperativity to *C*=2.5, as already obtained in ref. 25, and lowering the optical losses so that *κ*_1_/*κ*=0.9, for example, using an adiabatic cavity design^34^, would allow the ideal state orthogonality to be achieved with a mode reflectivity *R*_m_=65%. Further device optimization, through technological improvement of both cavity losses and QD dephasing, would allow approaching *R*_m_*=*1 in an ideal cavity-QED device (*C>>*1 and *κ*_*1*_/*κ=*1). In the framework of a future solid-state quantum network, performing novel spin–photon operations was previously envisioned in purely theoretical proposals^9^,^10^,^11^,^12^,^13^,^14^,^15^,^16^,^17^,^18^. It now becomes possible with the current technological state of the art.

## Methods

### Device fabrication

A GaAs/Al_0.9_Ga_0.1_As microcavity embedding self-assembled InAs QDs is grown by molecular beam epitaxy on a GaAs substrate. The bottom and top Bragg mirrors have 24 and 20 pairs, respectively, and present equal reflectivities (that is, *κ*_1_=*κ*_2_). The *in-situ* lithography technique is implemented to define pillars centred on single QDs. A red laser beam is first used to excite and monitor the QD emission, so as to measure its spatial position with 50-nm accuracy. A green laser beam is then used to expose, at the QD position, a layer of photoresist, which is subsequently used as a mask for inductively coupled plasma etching of the micropillar. The device selected in this work is charged with a resident hole and has a diameter of 2.1 μm.

### Resonant excitation experiments

The sample is maintained at 20 K inside a helium-vapour cryostat, together with an aspheric lens and cryogenic nanopositioners. The reflectivity is deduced from the reflected and incident powers measured with avalanche photodiodes and then normalized to unity when the laser photon energy is far from the cavity mode resonance. No magnetic field has been applied. Spin initialization and Kerr rotation measurements were performed using liquid-crystal variable waveplates, to compensate the polarization distortions induced by the various components along the optical paths.

### Identification of the carrier type

Although a single QD line is observed under resonant excitation measurements (as in Fig. 1e), four emission lines are observed in photoluminescence measurements with non-resonant excitation: neutral exciton, biexciton, negatively- and positively-charged trions. The exciton and biexciton lines are first identified by photon correlation measurements showing the characteristic bunching of the radiative cascade. Then, a definite proof that the line studied in Fig. 1e corresponds to the positively charged trion transition, and not the negatively charged one, is provided by the spin initialization experiment. Indeed, a hole spin can be initialized without any applied magnetic field, an effect that is not observed with an electron spin because of the strong electron–nuclei interaction, leading to short electron spin lifetimes *T*_1_^(electron)^≪*T*_1_^(hole)^ 23.

## Author contributions

C.A. and J.D. performed the experiments and analysed the experimental data. L.L. developed the theory of spin initialization and Kerr rotation in a cavity-QED device, with the help of M.G. V.L. participated in the experimental developments. A.L., I.S. and P.S. fabricated the sample. L.L. and P.S. conducted the project. All authors participated in scientific discussions and manuscript preparation.

## Additional information

**How to cite this article:** Arnold, C. *et al*. Macroscopic rotation of photon polarization induced by a single spin. *Nat. Commun.* 6:6236 doi: 10.1038/ncomms7236 (2015).

## Supplementary Material

Supplementary Information Supplementary Notes 1-3 and Supplementary References
