# Supplementary material for: Macroscopic rotation of photon polarization induced by a single spin
Source: Nat Commun. 2015 Feb 17;6:6236. doi: 10.1038/ncomms7236 (PMC4339913; doi:10.1038/ncomms7236)
Supplement: Supplementary Information — Supplementary Notes 1-3 and Supplementary References [file ncomms7236-s1.pdf]

SUPPLEMENTARY INFORMATION

Supplementary Note 1: Theoretical background

The master equation describing our cavity-QED device is of the following form: [1]

$$\dot{\rho} = -i[H, \rho] + \sum_j L_j(\rho), \qquad (1)$$

with ρ being the full density matrix, H being the Hamiltonian operator and L_j being the Lindblad superoperators for the different dissipative mechanisms.

Coherent processes – Hamiltonian

In the rotating wave approximation at laser energy ω (ħ = 1 units), the Hamiltonian operator is given by:

$$\begin{aligned}\hat{H} = (\omega_{QD} - \omega).\left(\hat{\sigma}_L^+\hat{\sigma}_L^- + \hat{\sigma}_R^+\hat{\sigma}_R^-\right) + (\omega_c - \omega).\left(\hat{a}_L^+\hat{a}_L + \hat{a}_R^+\hat{a}_R\right) + ig\left(\hat{a}_L^+\hat{\sigma}_L^- - \hat{\sigma}_L^+\hat{a}_L + \hat{a}_R^+\hat{\sigma}_R^- - \hat{\sigma}_R^+\hat{a}_R\right) \\ - i\sqrt{\kappa_1}\left[b_L^{in}\cdot(\hat{a}_L^+ - \hat{a}_L) + b_R^{in}\cdot(\hat{a}_R^+ - \hat{a}_R)\right] + (1/2)\cdot(\omega_X - \omega_Y)\cdot\left(\hat{a}_L^+\hat{a}_R + \hat{a}_R^+\hat{a}_L\right)\end{aligned} \qquad (2)$$

Here $\hat{a}_L$, $\hat{a}_R$ (resp. $\hat{a}_L^+$, $\hat{a}_R^+$) are annihilation (resp. creation) operators for L and R circularly-polarized photons in the cavity mode. Operator $\hat{\sigma}_L^-$ (resp. $\hat{\sigma}_L^+$) denotes the lowering (resp. raising) operator for the |⇑> - |⇑⇓↑> transition. Correspondingly, operator $\hat{\sigma}_R^-$ (resp. $\hat{\sigma}_R^+$) denotes the lowering (resp. raising) operator for the |⇓> - |⇓⇑↓> transition (see Fig. 2d for a scheme of the corresponding 4-level system and optical selection rules).

With these notations:

- The first Hamiltonian term describes the two quantum dot (QD) transitions |⇑> - |⇑⇓↑> and |⇓> - |⇓⇑↓>, characterized by the QD transition energy $\omega_{QD.}$
- The second term describes the energies of L- and R-polarized photons in the cavity mode, characterized by the cavity mode energy $\omega_{C.}$
- The third term describes the light-matter interaction for both optical transitions, characterized by the coupling strength *g*.
- The fourth term describes the driving field, characterized by the input fields b_L^{in} and b_R^{in} for the L- and R-polarized photons, and by κ_1 the top-mirror damping rate [2].
- The fifth term describes the internal exchange between L- and R-polarized intracavity photons, due to the slightly elliptical shape of the micropillar. This exchange is characterized by the polarization splitting ω_X - ω_Y, where ω_X and ω_Y are the energies of the two eigenmodes. With these notations the energy ω_C previously defined is given by $\omega_C = (\omega_X + \omega_Y)/2$.

Dissipative processes – Lindblad operators

The dissipative mechanisms are taken into account with Lindblad superators L_j in the following form:[1]

$$L_j(\rho) = (\Gamma_j / 2)\cdot\left(2O_j\rho\hat{O}_j^+ - \hat{O}_j^+\hat{O}_j\rho - \rho\hat{O}_j^+\hat{O}_j\right), \qquad (3)$$

with Γ_j and $\hat{O}_j$ the transition rates and the collapse operators for the different mechanisms. The processes involved are:

- Cavity damping for the L- and R-polarized photons: rate κ, collapse operators $\hat{a}_L$ and $\hat{a}_R$.
- Trion decay for the |⇑>-|⇑⇓↑> and |⇓>-|⇓⇑↓> transitions: rate γ_{decay}, collapse operators $\hat{\sigma}_L^-$ and $\hat{\sigma}_R^-$.
- Pure dephasing for the |⇑>-|⇑⇓↑> and |⇓>-|⇓⇑↓> transitions: rate $2\gamma^*$, collapse operators $\hat{\sigma}_L^+\hat{\sigma}_L^-$ and $\hat{\sigma}_R^+\hat{\sigma}_R^-$.[3]
- Hole spin-flip: rate $1/T_1^{(\text{hole})}$, collapse operators |⇑><⇓| and |⇓><⇑|
- Trion spin-flip: rate $1/T_1^{(\text{trion})}$, collapse operators |⇑⇓↑><⇓⇑↓| and |⇓⇑↓><⇑⇓↑|

With these notations the total QD dephasing rate γ is the sum of a life-time limited contribution and of a pure dephasing contribution, i.e:[4]

$$\gamma = \frac{\gamma_{decay}}{2} + \gamma^* , \tag{4}$$

where we assumed that $T_1^{(\text{hole})}$ and $T_1^{(\text{trion})}$ are much longer than the dephasing time γ^{-1}. We point out that γ_{decay} is mainly governed by the spontaneous emission of photons outside the fundamental cavity mode. It does not include the Purcell-accelerated emission of photons inside the cavity mode (at rate $2g^2/\kappa$), arising from the coherent interaction between the quantum dot and the confined mode, and described by the Hamiltonian above.

Since we do not observe any signature of phonon-sideband phenomena in our experimental data, and because the pure dephasing approach allows accounting for the phonon-induced dephasing of the zero-phonon line, the complete treatment of the QD-phonon interaction is not included in our model. We also note that while we use a pure-dephasing approach to describe charge-induced dephasing, a spectral diffusion approach can also successfully describe our observations. There is actually no clear way to discriminate between pure dephasing and charge-induced spectral diffusion in our experimental data.

Calculation tools

Our simulations are based on the resolution of the Lindblad master equation with a numerical quantum toolbox[5]. Because we are simulating experiments in the continuous-wave regime, predicted physical quantities are deduced from the stationary solution of the quantum master equation, $\dot{\rho} = 0$. The stationary density matrix then contains information on the populations and coherences of the four system states (including spin pumping and nonlinear optics processes), as well as information on the internal photonic field for each polarization. Using the input-output formalism,[2,6,7] one can then deduce the reflection coefficients and finally the polarization properties of the reflected photons.

Among the measurable quantities that one can retrieve using this technique, the complex reflection coefficients r_R and r_L, are crucial. Indeed, they allow determining the total mode reflectivity $R_m = |r_R|^2 + |r_L|^2$, but also the polarization properties of the reflected beam. This is described in Supplementary Note 3

Supplementary Note 2: Discussion on the system parameters

Estimations of the input coupling and of the critical photon number

There are two main quantities governing the nonlinear power-dependence of the QD resonance peak using linearly-polarized excitation (see Figs. 2a and 2c): the input-coupling efficiency and the critical photon number. In this section we describe how they can be estimated from the nonlinear behaviour of the QD resonance peak.

The method we use below has been previously validated with both continuous-wave and pulsed measurements in a previous work (See Ref. 7 and its Supplemental Materials for details).

On the one hand, the *input-coupling efficiency* η_{in} is given by the overlap between the spatial shapes of the free-space incident optical beam and of the confined cavity mode; it indicates the probability that an incident photon will indeed be coupled into the cavity. The average number of intracavity photons, denoted n, depends on the input-coupling efficiency η_{in}, on the incident optical power P_0, on the top-mirror damping rate κ_1, on the photon energy $\hbar\omega$, and on the complex reflection coefficient r. More precisely:

$$n = \frac{\eta_{in} P_0 \left|1-r\right|^2}{\kappa_1 \hbar \omega} \tag{5}$$

On the other hand, the *critical intracavity photon number* n_C gives the value of n for which the trion states become significantly populated. This critical intracavity photon number is defined as: [4,7]

$$n_C = \frac{\gamma \gamma_{decay}}{4 g^2} \tag{6}$$

The knowledge of n_C is strongly related to the knowledge of γ_{decay}, because the other parameters involved (g and γ) are already constrained by other experimental observations (QD peak amplitude and width). From typical lifetime measurements with similar quantum dots, we estimate that γ_{decay} is of the order of 1 ± 0.2 μeV (that is, a typical decay time of 600 ± 100ps). This gives an estimated value $n_C = 2.2\ 10^{-4}$, which is compatible with the observed nonlinear threshold if η_{in} = 75 ± 15%. This imperfect input coupling is related to an imperfect spatial overlap between the free space incident beam and the cavity mode, but also to the presence of a partially-etched nitride layer on the top of the pillar surface (this residual layer could not be removed without damaging the device). We point out that the uncertainty on γ_{decay} implies a corresponding uncertainty on the value of η_{in} : this makes it difficult to precisely evaluate η_{in} with only CW measurements. In Ref. 7, both CW and pulsed measurements had been performed and this indeterminacy could be reduced, allowing more precise values of η_{in} and γ_{decay} to be retrieved.

Estimation of the top-mirror output-coupling efficiency

In the absence of QD transition the mode reflectivity R_m is governed by the top-mirror output-coupling efficiency κ_1/κ, and at resonance the minimal value $R_m(\omega=\omega_c) = (1 - 2\ \kappa_1/\kappa)^2$ is obtained. This could be used to directly estimate κ_1/κ,; however, in our situation where the input-coupling is not perfect, the measured experimental reflectivity R_{exp} is not the exact mode reflectivity R_m. Indeed a spectrally-flat contribution is added to the reflectivity spectrum R_{exp}, coming from the reflected uncoupled light. This leads to an uncertainty on the intrinsic mode reflectivity $R_m(\omega=\omega_c)$, and thus to an uncertainty on κ_1/κ,. Because $\kappa_1 = \kappa_2$, as predicted by numerical simulations for a double-sided cavity with 20/24 (top/bottom) pairs, this uncertainty on κ_1 also leads to an uncertainty on κ_2, and finally to an uncertainty on $\kappa_s = \kappa - \kappa_1 - \kappa_2$.

In the extreme case where 100% input-coupling is achieved, $R_m = R_{exp}$, and the observed non-zero reflectivity at resonance is solely limited by the imperfect top-mirror output-coupling efficiency. This would correspond to $\kappa_1/\kappa = \kappa_2/\kappa$ = 26%, and thus a side-leakage contribution κ_s/κ = 48%. Another extreme case would be to have $R_m(\omega=\omega_c) = 0$ at resonance, which would correspond to a non-lossy device where $\kappa_s/\kappa = 0$, and thus $\kappa_1/\kappa = \kappa_2/\kappa$ = 50%. In this scenario the measured non-zero reflectivity at resonance is solely accounted for by the reflected uncoupled light. Our estimation that $\kappa_1/\kappa = \kappa_2/\kappa$ = 40 ± 5 %, and thus κ_s/κ = 20 ± 10 %, lies between these two extreme cases; it is also consistent with the side-leakage contribution expected in a 2.1 μm

diameter pillar. Last, but not least, these values produce predictions for the Kerr rotation angle which are in satisfying agreement with the observed experimental data.

Supplementary Note 3 : Polarization state for the reflected photons

Complex reflection coefficients in the low-power regime

The mode reflection coefficients for both R- and L-polarized photons depend on the spin state. In the low-power limit, and in the simple case where the mode polarization splitting can be neglected ($\omega_X = \omega_Y = \omega_C$), they are given by the following expressions: [2,8,9,10,11]

- If the spin is ⇓:

$$r_L(\Downarrow) = 1 - 2\frac{\kappa_1}{\kappa}\frac{1}{1 - i\Delta + \frac{2C}{1 - i\Delta'}} \quad (7)$$

and

$$r_R(\Downarrow) = 1 - 2\frac{\kappa_1}{\kappa}\frac{1}{1 - i\Delta} \quad (8)$$

- If the spin is ⇑:

$$r_L(\Uparrow) = 1 - 2\frac{\kappa_1}{\kappa}\frac{1}{1 - i\Delta} \quad (9)$$

and

$$r_R(\Uparrow) = 1 - 2\frac{\kappa_1}{\kappa}\frac{1}{1 - i\Delta + \frac{2C}{1 - i\Delta'}} \quad (10)$$

Note that the overall phase in these expressions is omitted, and that we have also neglected the imperfect coupling of the incident radiation with the cavity mode (see below).

These expressions involve the two figures of merit of our spin-photon interface:
- The cooperativity $C = g^2/\kappa\gamma$
- The top-mirror output coupling efficiency κ_1/κ

In addition, the reflection coefficients depend on two experimentally tuneable parameters:
- The normalized laser-cavity detuning: $\Delta = 2(\omega - \omega_C) / \kappa$
- The normalized laser-QD detuning: $\Delta' = (\omega - \omega_{QD}) / \gamma$

In this work the mode polarization splitting can not be neglected: we characterize it by the dimensionless normalized splitting $\delta = (\omega_X - \omega_Y) / \kappa$. Using the input-output formalism we obtain the following analytical formulas in the low-power limit:

$$r_L(\Downarrow) = r_R(\Uparrow) = 1 - 2\frac{\kappa_1}{\kappa}\frac{1 - \frac{i\delta}{1 - i\Delta}}{1 - i\Delta + \frac{2C}{1 - i\Delta'} + \frac{\delta^2}{1 - i\Delta}} \quad (11)$$

and:

$$r_R(\Downarrow) = r_L(\Uparrow) = 1 - 2\frac{\kappa_1}{\kappa}\frac{1 - \dfrac{i\delta}{1 - i\Delta + \dfrac{2C}{1 - i\Delta'}}}{1 - i\Delta + \dfrac{\delta^2}{1 - i\Delta + \dfrac{2C}{1 - i\Delta'}}} \tag{12}$$

These analytical expressions are in agreement with the numerical predictions obtained with the quantum toolbox. When $\delta = 0$ the above-mentioned expressions of r_L and r_R are retrieved. But in the presence of a cavity polarization splitting, the R- and L-polarizations are not eigenmodes of the system, so that additional terms appear describing the conversion between the circular polarizations.

Scalar product between the output polarization states

Starting from an input polarization state $(|L\rangle + |R\rangle)/\sqrt{2}$, the fact that r_L and r_R depend on the spin state leads to two different output polarization states, $|\Psi_\Uparrow\rangle$ and $|\Psi_\Downarrow\rangle$, for the spin states $|\Uparrow\rangle$ and $|\Downarrow\rangle$.:

$$\left|\Psi_\Downarrow\right\rangle = \frac{r_L(\Downarrow)\left|L\right\rangle + r_R(\Downarrow)\left|R\right\rangle}{\sqrt{\left|r_L(\Downarrow)\right|^2 + \left|r_R(\Downarrow)\right|^2}} \tag{13}$$

and: $$\left|\Psi_\Uparrow\right\rangle = \frac{r_L(\Uparrow)\left|L\right\rangle + r_R(\Uparrow)\left|R\right\rangle}{\sqrt{\left|r_L(\Uparrow)\right|^2 + \left|r_R(\Uparrow)\right|^2}} \tag{14}$$

With these notations the scalar product between these two states is simply written as:

$$\left\langle \Psi_\Downarrow \middle| \Psi_\Uparrow \right\rangle = \frac{r_L(\Downarrow)r_L^*(\Uparrow) + r_R(\Downarrow)r_R^*(\Uparrow)}{\sqrt{\left|r_L(\Downarrow)\right|^2 + \left|r_R(\Downarrow)\right|^2}\sqrt{\left|r_L(\Uparrow)\right|^2 + \left|r_R(\Uparrow)\right|^2}} \tag{15}$$

As discussed in the main text, if the scalar product. $\langle\Psi_\Downarrow|\Psi_\Uparrow\rangle$ reaches zero a perfect quantum non-demolition measurement[12] becomes possible, conditioned by the detection of a single reflected photon. This state orthogonality $\langle\Psi_\Downarrow|\Psi_\Uparrow\rangle=0$ can be reached for a large range of device parameters (C and κ_1/κ), as shown in Fig. 4b, by properly adjusting the normalized laser-cavity and laser-QD detunings Δ and Δ'. Contrary to previous theoretical predictions with ideal systems[10], this possibility is here achievable with realistic devices. The counterpart is that, for the chosen values of Δ and Δ' allowing $\langle\Psi_\Downarrow|\Psi_\Uparrow\rangle$ to reach zero, the mode reflectivity $R_m = |r_R|^2 + |r_L|^2$ will not equal unity. If single-photon sources are used to excite the device, this will limit the success rate of the spin-photon interface, together with the imperfections of the single-photon sources, detectors, optical couplings, etc… this limitation is not strong, however, as large values of the mode reflectivity are achievable with realistic devices (Fig. 4c).

Supplementary References

[1] H. Carmichael, *An Open Systems Approach to Quantum Optics*, Springer (1994)
[2] D. Walls and G. Milburn, *Quantum Optics*, Springer (1994)
[3] A. M. Zagoskin, *Quantum Engineering*, Cambridge University Press (2011)
[4] M. A. Armen and H. Mabuchi, Physical Review A 73, 063801 (2006)
[5] S. Tan, Journal of Optics B: Quantum and Semiclassical Optics 1, 424 (1999)

[6] E. Waks and J. Vuckovic, Phys. Rev. Lett. 96, 153601 (2006)
[7] V. Loo et al, Physical Review Letters 109, 166806 (2012)
[8] L. Lugiato, *Theory of Optical Bistability,* Progress in Optics XXI, Elsevier (1984)
[9] C. Y. Hu, W. J. Munro and J. G. Rarity, Phys. Rev B 78, 125318 (2008)
[10] C. Y. Hu, A. Young, J. L. O'Brien, W. J. Munro, and J. G. Rarity, Phys. Rev. B 78, 085307 (2008)
[11] C. Bonato et al, Phys. Rev. Lett.104, 160503 (2010)
[12] S. Haroche and J.M. Raymond, *Exploring the Quantum: Atoms, Cavities and Photons,* Oxford Graduate texts (2006)
